# Detecting Macular Disease Based on Optical Coherence Tomography Using a Deep Convolutional Network

**DOI:** 10.3390/jcm12031005

**Published:** 2023-01-28

**Authors:** Jinyoung Han, Seong Choi, Ji In Park, Joon Seo Hwang, Jeong Mo Han, Junseo Ko, Jeewoo Yoon, Daniel Duck-Jin Hwang

**Affiliations:** 1 Department of Applied Artificial Intelligence, Sungkyunkwan University, Seoul 03063, Republic of Korea; 2 Department of Human-Artificial Intelligence Interaction, Sungkyunkwan University, Seoul 03063, Republic of Korea; 3 RaonData, Seoul 04615, Republic of Korea; 4 Department of Medicine, Kangwon National University Hospital, Kangwon National University School of Medicine, Chuncheon 24341, Gangwon-do, Republic of Korea; 5 Seoul Plus Eye Clinic, Seoul 01751, Republic of Korea; 6 Seoul Bombit Eye Clinic, Sejong 30127, Republic of Korea; 7 Department of Ophthalmology, Hangil Eye Hospital, #35 Bupyeong-daero, Bupyeong-gu, Incheon 21388, Republic of Korea; 8 Department of Ophthalmology, Catholic Kwandong University College of Medicine, Incheon 22711, Republic of Korea; 9 Lux Mind, Incheon 21388, Republic of Korea

**Keywords:** deep learning, retinopathy, convolutional neural network, medical image

## Abstract

Neovascular age-related macular degeneration (nAMD) and central serous chorioretinopathy (CSC) are two of the most common macular diseases. This study proposes a convolutional neural network (CNN)-based deep learning model for classifying the subtypes of nAMD (polypoidal choroidal vasculopathy, retinal angiomatous proliferation, and typical nAMD) and CSC (chronic CSC and acute CSC) and healthy individuals using single spectral–domain optical coherence tomography (SD–OCT) images. The proposed model was trained and tested using 6063 SD–OCT images from 521 patients and 47 healthy participants. We used three well-known CNN architectures (VGG–16, VGG–19, and ResNet) and two customized classification layers. Additionally, transfer learning and mix–up-based data augmentation were applied to improve robustness and accuracy. Our model demonstrated high accuracies of 99.7% and 91.1% in the nAMD and CSC classification and retinopathy (nAMD and CSC) subtype classification, including normal participants, respectively. Furthermore, we performed an external test to compare the classification accuracy with that of eight ophthalmologists, and our model showed the highest accuracy. The region determined to be important for classification by the model was confirmed using gradient-weighted class activation mapping. The model’s clinical criteria were similar to that of the ophthalmologists.

## 1. Introduction

Neovascular age-related macular degeneration (nAMD) and central serous chorioretinopathy (CSC) are common retinopathies in patients [1]. In developed countries, nAMD, a degenerative macular disease, is the leading cause of blindness in individuals aged >50 years [2]. The three subtypes of nAMD are polypoidal choroidal vasculopathy (PCV), retinal angiomatous proliferation (RAP), and typical nAMD [3]. CSC can be classified as chronic and acute CSCs, depending on the severity of separation of the neurosensory retina in the posterior pole [4]. Furthermore, many patients with CSC are characterized by reduced and/or distorted vision with altered color sensitivity [5]. Compared with acute CSC, chronic CSC often requires active intervention because of irreversible poor vision. Therefore, diagnosing CSC and determining its chronicity is essential for a future treatment plan or predicting a prognosis [1].

Regarding macular diseases, an accurate diagnosis is vital because each retinopathy subtype has significantly different prognoses and treatment strategies. Therefore, ophthalmologists use several modalities, including fluorescein angiography (FA), indocyanine green angiography (ICGA), fundus autofluorescence (AF), and optical coherence tomography (OCT).

OCT is the primary modality used recently to diagnose structural abnormalities associated with nAMD and CSC. Progress in deep learning techniques, such as convolutional neural networks (CNNs) has enabled the classification of nAMD, CSC, and other retinopathies using OCT images [6,7,8,9]. Hwang et al. [6] reported that distinguishing RAP from PCV using a deep learning model and OCT images is possible. Yoon et al. [7] applied a deep learning approach to distinguish between chronic and acute CSC using OCT. In addition, a study was conducted to classify several macular diseases simultaneously using two modalities (that is, PCV vs. nAMD/PCV vs. nAMD vs. dry AMD vs. normal AMD) [10,11].

As mentioned above, subtype classification of macular diseases is important; however, in actual clinical practice, ophthalmologists may experience difficulty diagnosing nAMD and CSC because of their structural similarities. In particular, if nAMD is incorrectly diagnosed as CSC, it can lead to blindness in severe cases, and treatment approaches should differ between the two retinopathies. Therefore, it is clinically important to classify nAMD and CSC simultaneously and accurately into subtypes. To our knowledge, no studies on the automated classification of nAMD and CSC subtypes exist.

In this study, we proposed a deep learning model that classifies representative retinal diseases, nAMD and CSC, and normal groups simultaneously (3-class classification), and additionally classifies five subtypes of nAMD, CSC, and normal groups simultaneously (6-class classification). To generate a general comprehensive diagnosis model, we adopted the model with the highest performance using three CNN-based models (VGG–16, VGG–19 [12], and ResNet [13]) with two custom layers. To train our model using a small spectral–domain OCT (SD–OCT) dataset, we applied transfer learning using ImageNet [14] and mix–up [15] data augmentation.

## 2. Materials and Methods

### 2.1. Data Collection and Labeling

The dataset was collected from the medical records of patients who visited the Hangil Eye Hospital between 2014 and 2020. Our study used SD–OCT (Heidelberg Spectralis; Heidelberg Engineering, Heidelberg, Germany) images of healthy participants and patients with nAMD (PCV, RAP, and typical nAMD) or CSC (acute and chronic CSC). As shown in Table 1, the entire SD–OCT dataset comprised 6063 SD–OCT images from 521 patients and 47 healthy participants. Of the 521 patients, 330 had nAMD, with 115, 101, and 114 having PCV, RAP, and typical nAMD, respectively. A total of 723 SD–OCT images were obtained from 88 chronic cases, and 882 were obtained from 103 acute cases, accounting for a total of 191 patients with CSC. Two retinal specialists with over 10 years of clinical experience verified the SD–OCT dataset based on various data, including fundus examinations, FA, ICGA, and the patients’ medical records. Another retinal specialist evaluated the discrepancy in cases where the two specialists had different opinions. Any discrepancies were resolved by consensus.

### 2.2. SD–OCT Dataset Collection

**Figure 1:**
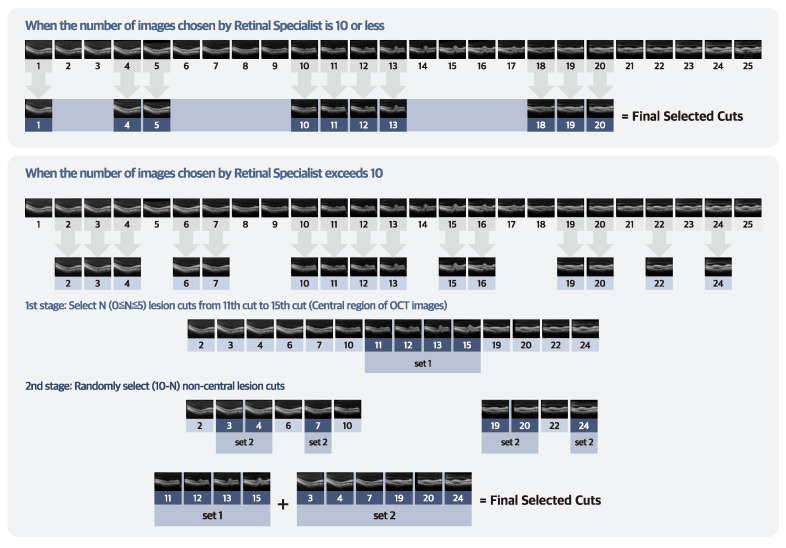
The extraction process of lesion cuts from 25 SD–OCT scan images. The retina specialists initially extracted lesion cuts from each patient’s SD–OCT images. If the extracted lesion cuts from a patient were ≤10, all lesion cuts were used as SD–OCT datasets. However, if the total number of lesions cut was >10, we selected N (that is, 0 ≤ N ≤ 5) lesion cuts between the 11th–15th central regions. Finally, we randomly selected 10–N lesion cuts from non–centered regions (1st–10th and 16th–25th). In this study, we selected about 10 images per patient.

Figure 1 illustrates the overall process of extracting the lesion cuts for each patient from the 25 scanned OCT images. The retinal specialists with over 10 years of clinical experience carefully selected the lesion cut by viewing all 25 SD–OCT images with the following criteria: (1) subretinal fluid (SRF), (2) intraretinal fluid (IRF), (3) irregular RPE elevation with double layer sign, (4) pigment epithelial detachment (PED), and (5) subretinal hyperreflective material.

Subsequently, we selected N (that is, 0 ≤ N ≤ 5) lesion cuts in the central region (between the 11th–15th cuts). Then, we randomly selected 10–N non–central lesion cuts positioned between the 1st–10th cuts and between the 16th–25th cuts (including the parafoveal or perifoveal area). We selected all non–central lesion cuts when their number was < 10–N. About 10 images per patient were selected for this study.

### 2.3. Experimental Setup

To train and evaluate the proposed model, we split the entire SD–OCT dataset into training (80%), validation (10%), and testing (10%) sets. The training set consisted of 4878 images (normal, 1650; PCV, 729; RAP, 637; typical nAMD, 603; chronic CSC, 578; acute CSC, 681) of 412 patients (PCV, 90; RAP, 80; typical nAMD, 90; chronic CSC, 71; acute CSC, 81) and 37 healthy participants. The test set consisted of 596 images (normal, 175; PCV, 104; RAP, 85; typical nAMD, 80; chronic CSC, 55; acute CSC, 97) of 54 patients (PCV, 13; RAP, 10; typical nAMD, 12; chronic CSC, 8; acute CSC, 11) and five healthy participants. All 589 SD–OCT datasets not used in the training and test sets were used as the validation datasets. To precisely measure the model’s performance, we constructed a dataset that did not include the SD–OCT images of the same patient in the training, validation, and test sets simultaneously.

To fairly compare all possible model architectures, all the models were trained using the same hyperparameters. The batch size of the model was 64, the epochs were 100, the loss function was the categorical cross-entropy with Adam optimization [16], and the learning rate was 0.0001.

An external test was conducted by eight ophthalmologists to compare the classification accuracy and evaluate the proposed model from a clinical perspective. The external test dataset consisted of 379 SD–OCT images (normal: 150, PCV: 47, RAP: 42, typical nAMD: 52, chronic CSC: 41, and acute CSC: 47). The external test dataset included only patients who were not included in the training, validation, and test sets. The eight ophthalmologists consisted of three residents, three fellows, and two retinal specialists with more than 10 years of clinical experience. The ophthalmologists classified the external test dataset using a web–based test tool designed for this experiment. The test tool is designed for ophthalmologists to view a single SD–OCT image and select whether it is normal, nAMD (PCV, RAP, or typical nAMD), or CSC (chronic or acute CSC). We also measured kappa coefficients [17] to determine the similarity of the proposed model to the classification criteria of the two retina specialists.

### 2.4. Data Augmentation

We applied data augmentation during the training phase because of the lack of large-scale SD–OCT training data. Data augmentation has been proven effective in enhancing classification tasks’ performances [6,7]. In particular, we used a mix–up [15] algorithm that combines two images to generate a new training set based on the combined images. A newly created image can be calculated as follows:

(1) $$\tilde{x}=\lambda x_{i}+\left(1-\lambda \right)x_{j}$$

(2) $$\tilde{y}=\lambda y_{i}+\left(1-\lambda \right)y_{j}$$

where (*x_i_*, *y_i_*) and (*x_j_*, *y_j_*) are two randomly selected examples from the training dataset, and λ is a randomly assigned value ranging between 0–1. By repeating this, the mix–up extends the training distribution by incorporating prior knowledge that linear interpolations of feature vectors should lead to linear interpolations of the associated targets.

### 2.5. Model Architecture

**Figure 2:**
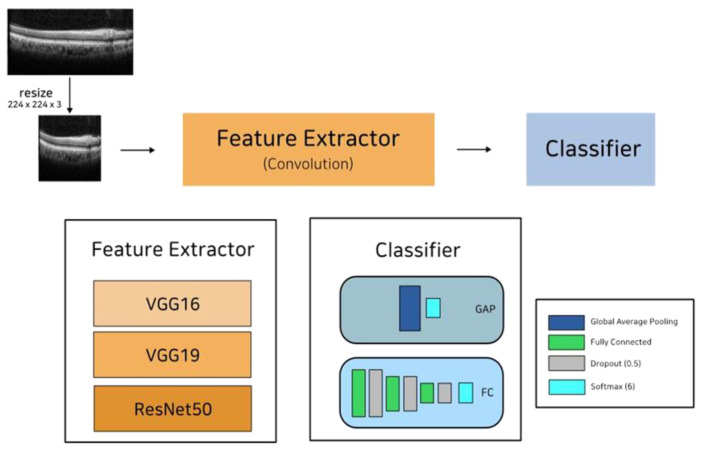
The input image is an SD–OCT image. First, the input image is resized to 224 × 224 × 3. Then, the resized image is given to the feature extractor layer. Finally, features extracted using the feature extractor are passed to the classifier layer for final classification.

To build an accurate image classification architecture, we first evaluated well-known CNN models: VGG–16, VGG–19 [12], and ResNet [13]. Then, to remedy the shortage of training data, we applied transfer learning using the ImageNet dataset [14]. ImageNet has 15 million annotated images with 1000 classes. We obtained an accurate and robust model by transferring the ImageNet–based pre–trained model to SD–OCT images. For each CNN model, the fully connected layers of the original models (VGG–16, VGG–19, and ResNet) were replaced with two custom settings: (1) four fully connected layers and three dropout layers with leaky ReLU [18] as the activation function and (2) a global average pooling layer. Figure 2 illustrates the architecture of the proposed model.

## 3. Results

### 3.1. Model Performance

In this study, we first conducted experiments to compare three different CNN models (VGG–16, VGG–19 [12], and ResNet [13]) with two custom settings (fully connected layers and global average pooling). Then, we replaced the fully connected layers of the original CNN models with two custom layers. In particular, fully connected layers were composed of four dense and three dropout layers, and the activation function was Leaky ReLU [18]. For the global average pooling layer, the features generated in the two-dimensional global average pooling layer pass through a dense layer. The activation function of the last dense layer used in the two custom layers was set to the softmax function. Finally, the model could classify nAMD, CSC, and normal groups (denoted by 3–class classification) and the subtypes of nAMD, CSC, and normal groups (denoted by 6–class classification).

**Figure 3:**
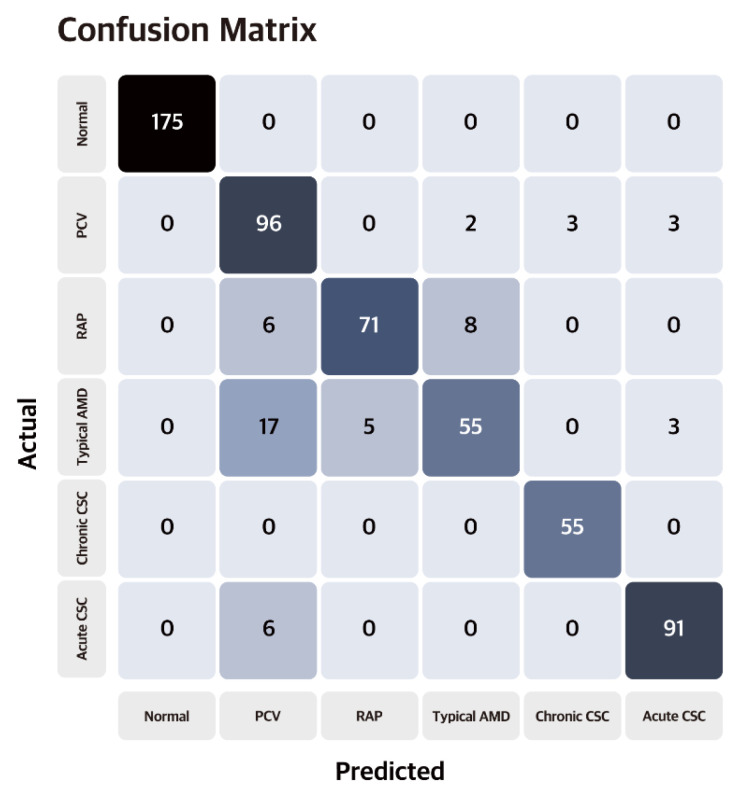
Confusion matrix of the proposed model. The vertical axis is the ground truth, and the horizontal axis is the model prediction. Our proposed model recorded 91.1% classification accuracy for the test set.

As shown in Table 2, the VGG–19-based model with four fully connected layers showed the highest accuracies (99.7%, 91.1%) in classifying normal, nAMD, and CSC groups (3–class classification) and the subtypes of normal, nAMD, and CSC groups (6–class classification), respectively. Figure 3 shows the retinopathy subtype classification model performed accurately.

### 3.2. Comparison with Ophthalmologists

**Figure 4:**
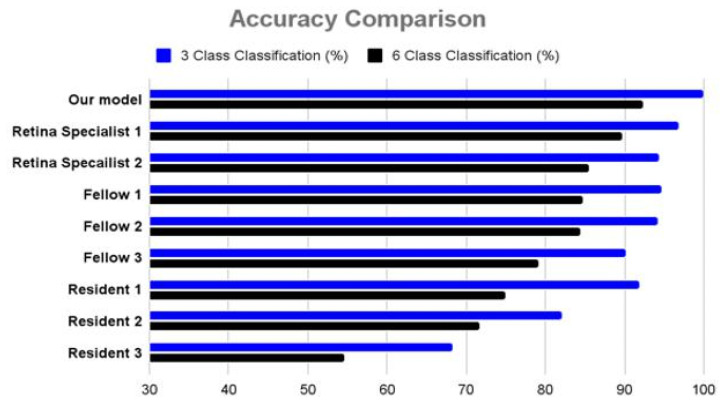
Comparison of classification performance between our model and eight ophthalmologists using an external test dataset. Our model’s classification accuracies were 100 and 92.3% in 3-class and 6-class classifications, respectively. In addition, our model recorded the highest performance compared with the eight ophthalmologists in both tests.

A classification test based on an external test dataset was performed to compare the classification accuracy of the proposed model with that of ophthalmologists. The external test dataset consisted of 379 SD–OCT images (normal: 150; PCV: 47; RAP: 42; typical nAMD: 52; chronic CSC: 41; acute CSC: 47) of 26 patients (PCV, 6; RAP, 5; typical nAMD, 6; chronic CSC, 4; acute CSCS, 5) and six healthy participants. A performance comparison between the proposed models and the eight ophthalmologists is shown in Figure 4. The average classification accuracies of the eight ophthalmologists were 88% (3-class classification) and 76% (6-class classification), respectively.

Of the eight ophthalmologists, retinal specialists with more than 10 years of clinical experience showed the highest classification accuracies, with 97.1 and 89.7% in 3-class and 6-class classifications, respectively, which were lower than the accuracies of the proposed model (100% in 3-class classification and 92.3% in 6-class classification). Furthermore, we measured the similarity of judgment between retinal specialists and the proposed model using kappa coefficients [17]. In the subtype classification, the kappa coefficient between the two retina specialists was 0.80, indicating that the judgment was almost similar. In addition, the kappa coefficients between our model and the two specialists were 0.76 and 0.82. Therefore, we confirmed that the decision-making criteria for classifying nAMD and CSC subtypes using the proposed model were similar to those of the specialists.

Furthermore, as shown in Table 3, the proposed model performed better than the two retina specialists in terms of the precision, recall, and F1–score metrics used for classification. In the subtype classification test (6–class classification), 18 cases in which more than five of the eight ophthalmologists made misclassifications were identified. However, the proposed model accurately classified all 18 cases in which professional experience diagnosing retinal disorders were required. Based on this result, our model can support the diagnosis of nAMD and CSC subtypes in actual clinical settings.

### 3.3. Gradient-Weighted Class Activation Mapping (Grad–CAM) Images

**Figure 5:**
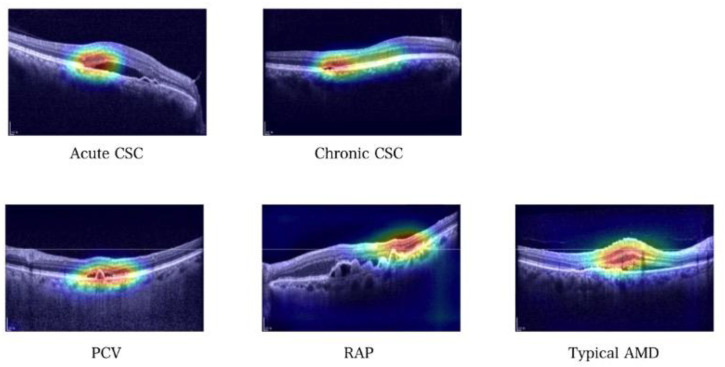
Grad–CAM visualization of five cases of nAMD and CSC subtypes. The high-intensity area (red and yellow color) reflects the area of interest in our model. The corresponding Grad–CAM image shows that our model focused on similar clinical criteria for classifying nAMD and CSC subtypes.

Gradient-weighted class activation mapping (Grad–CAM) [19] was applied to visualize the regions recognized by a deep learning-based model as important features for classification. The regions where the proposed model focused on SD–OCT images for classifying nAMD and CSC subtypes were visualized using Grad–CAM. The representative heat maps generated using Grad–CAM are shown in Figure 5. The areas highlighted in the heat map are the parts the model considers essential for classification tasks. These areas are similar to the regions ophthalmologists usually examine when diagnosing nAMD and CSC subtypes [7,20].

## 4. Discussion

This study established a deep learning-based model for distinguishing between various nAMD and CSC subtypes using SD–OCT images and evaluated its performance. The proposed model effectively classified the normal, nAMD, and CSC groups. Furthermore, it classified the nAMD groups into PCV, RAP, and typical nAMD and CSC groups into chronic and acute CSCs. Several deep learning-based methods have been applied to optimize the performance of the proposed model in classifying nAMD and CSC subtypes using a limited number of SD–OCT images. First, transfer learning was applied to reuse the knowledge of a source domain and solve the target task (classifying nAMD and CSC subtypes). In this study, a pre-trained model based on the ImageNet [14] dataset was trained using the SD–OCT dataset. Second, mix–up [15] data augmentation was used to generate a robust deep learning model and reduce overfitting by increasing the variance of the SD–OCT dataset. The mix–up approach generated a new training dataset by combining two SD–OCT images and labels by applying weights between 0–1, which were randomly selected. We achieved high classification accuracies and robust performance by combining these methods, even within a limited SD–OCT dataset.

Based on the external test dataset, our proposed model showed the highest classification accuracy compared with the eight ophthalmologists. For 3-class classification (nAMD, CSC, and normal groups), the proposed model achieved 100% classification accuracy. Furthermore, the proposed model showed the highest classification accuracy for 6-class subtype classification (PCV, RAP, typical nAMD, chronic CSC, acute CSC, and normal groups) compared with the eight ophthalmologists. In the subtype classification task, among the 379 SD–OCT images included in the external dataset, 18 that were misclassified by five ophthalmologists were accurately classified by the model. In addition, the kappa coefficient [17] between the two retinal specialists and our model was high (0.76, 0.82). This suggests that the proposed deep learning-based model can support ophthalmologists who are not retinal specialists in classifying various subtypes of nAMD and CSC, a task that requires the involvement of skilled retinal specialists.

Furthermore, based on Grad–CAM [19] images, the subtype classification criteria of the proposed model were observed. Based on the highlighted regions on the SD–OCT images, we showed that the proposed model learned according to clinically meaningful criteria [7,20]. The foveal region of the retina was primarily highlighted, indicating that our model focused on foveal lesions when differentiating between subtypes of nAMD/CSC. Notably, this is the region at which ophthalmologists mainly examine when classifying nAMD or CSC using OCT. When the model plays an auxiliary role in the diagnosis by ophthalmologists in actual clinical practice, more reliable interpretations can be made by ophthalmologists if visualization tools such as Grad-CAM are presented in addition to the model’s reading results.

This study had several limitations. First, the variety and number of available SD–OCT images were limited. All images were acquired using a single OCT device. In future studies, external validation using OCT devices sourced from different manufacturers is necessary. However, the dataset was sufficient to demonstrate the feasibility of the proposed deep learning-based model in distinguishing between various nAMD and CSC subtypes using OCT images. Second, the performance of the proposed model was evaluated using only a single OCT image. In clinical environments, ophthalmologists usually arrive at a comprehensive diagnosis by examining several OCT images obtained from a single patient. For an effective diagnosis of nAMD and CSC, combining multiple images would be better than using only a single OCT image. Third, this is a cross-sectional study. This model can be extended to predict disease progression using a series of OCT images. In addition to determining the status by observing the latest images, extended models can use longitudinal image data obtained from nAMD and CSC patients to predict future progression or response to treatment.

Regardless of the study’s limitations, the developed model demonstrates good and promising diagnostic performance and emphasizes the need for further investigations on its potential impact on the clinical diagnosis of nAMD and CSC. The proposed model can be clinically useful in determining treatment plans or predicting prognoses, depending on the subtypes of nAMD and CSC. In conclusion, we developed a deep learning model that effectively distinguished between various subtypes of nAMD and CSC using only OCT images. The deep learning-based model can help ophthalmologists distinguish between nAMD and CSC subtypes by automating the classification process. This study provides a basis for further research on the development of accurate OCT-based deep learning models that demonstrate enhanced performance in detecting nAMD and CSC subtypes and classifying several types of macular diseases.

## Figures and Tables

**Table 1:** Total SD–OCT dataset.

	Normal	nAMD			CSC		Total
		PCV	RAP	Typical nAMD	Chronic	Acute	
Image, no	1975	908	821	754	723	882	6063
Participants, no	47	115	101	114	88	103	568

**Table 2:** Comparative results of CNN-based models (VGG–16, VGG–19, and ResNet).

Base Model	Custom Layer	3 Class Classification	6 Class Classification
VGG–16	Fully Connected layer	99.1%	90.3%
	Global Average Pooling	94.1%	86.9%
VGG–19	Fully Connected layer	99.7%	91.1%
	Global Average Pooling	97.6%	86.1%
Resnet	Fully Connected layer	98.5%	87.4%
	Global Average Pooling	98.1%	85.4%

**Table 3:** Comparative results of classification performance of the proposed model and two retina specialists.

	Class	Precision	Recall	F1–Score
Proposed Model	Normal	1.00	1.00	1.00
	PCV	0.77	0.92	0.84
	RAP	0.93	0.84	0.88
	Typical nAMD	0.85	0.69	0.76
	Chronic CSC	0.95	1.00	0.97
	Acute CSC	0.94	0.94	0.94
Retina Specialist 1	Normal	0.93	0.91	0.92
	PCV	0.81	0.95	0.88
	RAP	1.00	1.00	1.00
	Typical nAMD	0.74	0.66	0.70
	Chronic CSC	0.92	0.86	0.89
	Acute CSC	0.76	0.79	0.77
Retina Specialist 2	Normal	0.88	0.81	0.84
	PCV	0.83	0.85	0.84
	RAP	0.98	0.99	0.98
	Typical nAMD	0.63	0.72	0.67
	Chronic CSC	0.94	0.69	0.79
	Acute CSC	0.64	0.71	0.67

## Data Availability

The data are not available for public access because of patient privacy concerns but are available from the corresponding author upon reasonable request.
